# NIPAm Microgels Synthesised in Water: Tailored Control of Particles’ Size and Thermoresponsive Properties

**DOI:** 10.3390/polym16243532

**Published:** 2024-12-18

**Authors:** Gabriela Rath, Davide Mazzali, Ali Zarbakhsh, Marina Resmini

**Affiliations:** School of Physical & Chemical Sciences, Queen Mary University of London, Joseph Priestley Building, Mile End Road, London E1 4NS, UK; g.rath@qmul.ac.uk (G.R.); d.mazzali@qmul.ac.uk (D.M.); a.zarbakhsh@qmul.ac.uk (A.Z.)

**Keywords:** polymeric microgels, NIPAm, free radical polymerisation, sustainability, thermoresponsive particles

## Abstract

Microgels, combining the properties of hydrogels and microparticles, are emerging as versatile materials for varied applications such as drug delivery and sensing, although the precise control of particle size remains a challenge. Advances in synthetic methodologies have provided new tools for tailoring of properties, however costs and scalability of the processes remains a limitation. We report here the water-based synthesis of a library of *N*-isopropylacrylamide-based microgels covalently crosslinked with varying contents of *N*,*N′*-methylenebisacrylamide. The results highlight the versatility of water as a synthetic medium, which yields large and monodisperse microgels, with excellent control over size. Dynamic light scattering data demonstrate that by increasing the total monomer concentration from 1 to 3 wt%, the particle size is increased by up to 4.9-fold. Crosslinker content allows fine-tuning of microgel size, with greater relevance for functionalised microgels. Functional co-monomers such as *N*-(3-aminopropyl)methacrylamide hydrochloride and *N*-(hydroxymethyl)acrylamide are shown to influence size and thermoresponsive behaviour, with hydrogen-bonding monomers reducing particle size and increasing the volume phase transition temperature by 2 °C. Positively charged monomers show a size reduction upon heating but provide colloidal stability at temperatures up to 60 °C. These findings emphasize the importance of tailoring synthetic conditions and formulations to optimize microgel properties for specific applications.

## 1. Introduction

The development of innovative materials for biomedical applications has been a key outcome of advances in nanotechnology, with applications in areas such as sensing [1], catalysis [2], and drug delivery [3,4]. However, the challenges associated with the scalability and sustainability of the synthetic methodology have limited their wider use on an industrial scale. Among the different materials studied, polymeric organic microgels have raised considerable interest, with their combined feature of hydrogels and microparticles [5], and properties such as the ability to form stable colloidal dispersions in different solvents [6,7], large specific surface areas [8], and easily tailored formulations [9,10,11]. Further, stimuli-responsive microgels are of great interest to the field of nanomedicine [12], with thermoresponsive polymers being targeted, given their ability to tailor drug release [13] above a certain critical temperature. Among the different monomers that yield thermoresponsive polymers, *N*-isopropylacrylamide (NIPAm) is certainly the most widely studied [14], as it shows a transition temperature close to the physiological one. This value can be controlled, and it is greatly influenced by the hydrophilicity of the resulting microgels [8]. However, challenges associated with the ability to control the particle size have limited the commercialisation of thermoresponsive microgels [15], as size has been shown to significantly influence, for example, the distribution [16,17] and interaction of these materials with biomolecules [6] within the body. Therefore, developing simple and scalable methods that allow rigorous control over parameters such as size and incorporation of functional monomers is crucial to expedite the applications of these materials in different fields. Growing interest in controlled/living polymerisation in recent years can be attributed to its enhanced control over microgel size, flexibility, and the possibility of obtaining complex architectures [18]. However, the significant costs and challenges of scaling up remain limiting factors for industrial applications [19]. In contrast, free radical polymerisation offers a simple, efficient, cheap, and scalable method, having been used to prepare almost half of all commercial synthetic polymers available today [20]. Frequently, free radical polymerisation requires organic solvents (e.g., toluene, acetone, and ethyl acetate) to obtain high polymerisation rates on an industrial scale. However, challenges associated with solvent handling, environmental impact, and safety considerations have driven changes towards using water as the synthetic solvent, resulting in several advantages [21,22]. Previous work explored the use of water in combination with organic solvents [23,24] or as the only solvent [25] to produce a variety of microgels, including stimuli-responsive microgels [14].

Our group has investigated free radical polymerisation, more specifically high dilution radical polymerisation (HDRP), to synthesize acrylamide-based nanogels, with a focus on how the chemical composition, matrix rigidity, and synthetic methodologies affect their thermoresponsive properties [7], surface activity [26], and interaction with biological molecules [27]. Recently, nanogels synthesised via free radical polymerisation showed good biocompatibility in vitro and in vivo (in zebrafish models) [28], making them strong candidates for drug delivery [29]. Initially synthesised in organic solvents, such as dimethylsulfoxide (DMSO), recent efforts have shifted towards more sustainable water-based methodologies [30].

While nanogels are ideal for systemic applications [31] requiring deep tissue penetration [32,33] or cellular uptake [34], microgels, with sizes comprised between 0.2 to 1 μm, offer specific advantages for localised treatments [35] and applications where larger cargo capacity [36,37] or mechanical stability [38] are essential, making them especially valuable in fields like regenerative medicine [39], localised drug delivery [40,41,42], and tissue engineering [43,44,45]. However, the impact of changing the crosslinker content and tailoring the formulation by incorporating different functional groups to control particle size and physicochemical properties of microgels has yet to be fully explored.

We report here our results on the synthesis via free radical polymerisation of NIPAm-based microgels covalently crosslinked with *N,N’*-methylenebisacrylamide (MBA), synthesised in water, to evaluate the impact of changing the synthetic parameters on the physicochemical properties of the microgels together with their thermoresponsive behaviour. The formulations were further tailored by adding *N*-(3-aminopropyl)methacrylamide hydrochloride (NAPMAm) and *N*-(hydroxymethyl)acrylamide (NHMAm) as functional monomers to investigate the effects of introducing positive charges and hydrogen-bonding groups on the particles’ size, using dynamic light scattering (DLS) and transmission electron microscopy (TEM). The crosslinker content was varied between 2 and 10 molar% to identify any correlation with size and properties. The thermoresponsive behaviour of the microgels was evaluated, and the volume phase transition temperature (VPTT) values were determined for each formulation using UV–vis spectrophotometry complemented by dynamic light scattering.

## 2. Materials and Methods

### 2.1. Materials

All materials were used as received unless stated otherwise. MBA (99%), NAPMAm (98%), cetrimonium bromide (CTAB, ≥99%), and 1,2,4,5-tetramethylbenzene (TMB, 98%) were purchased from Sigma-Aldrich (Gillingham, UK). NIPAm (97%) and azobisisobutyronitrile (AIBN, 98%) were purchased from Sigma-Aldrich and used after recrystallisation in hexane and methanol, respectively. NHMAm(98%) was purchased from Tokyo Chemical Industry UK Ltd. (Oxford, UK). Acetone was obtained from Fisher Scientific (Loughborough, UK). The dialysis membrane (regenerated cellulose, molecular weight cut-off: 3500 Da, width: 34 mm, diameter: 22 mm) was purchased from Medicell International Ltd. (London, UK).

### 2.2. Synthesis of Microgels in Water

Microgels were synthesised via free radical polymerisation in water following our previously reported procedure [7]. NIPAm, MBA, NAPMAm, and NHMAm were dissolved in the appropriate volume of water to yield a total monomer concentration (C_m_) ranging from 1 to 3 wt%. CTAB was solubilised in deionised water in a separate round-bottom flask and added to the reaction mixture at a final concentration of 0.6 mg mL^−1^. All flasks were sealed, purged with N_2_ for 45 min, and placed in an oil bath at 70 °C. AIBN (initiator) was solubilised in acetone and added to the reaction mixture at a concentration of 1.7 molar% of the total moles of double bonds present in the mixture. After 24 h, the reaction mixture was removed from the oil bath and allowed to cool down to room temperature before removing the rubber seal.

The resulting microgels’ solutions were purified by dialysis against deionised water for 3 days, changing water thrice a day. The isolated polymer was obtained by freeze-drying (LTE Scientific Lyotrap, Greenfield, UK), yielding a soft white powder that was stored at room temperature. Reported yields refer to the isolated microgels following freeze-drying.

### 2.3. Characterisation of Microgels

#### 2.3.1. Monomer Conversion via ^1^H-NMR

Aliquots (100 µL) of the polymerisation mixture were drawn immediately after monomer complete solubilization (time zero, t_0h_) and after the reaction was quenched (time 24 h, t_24h_) using a micro syringe. The aliquots were mixed with 400 µL of TMB (internal standard) stock solution (1.5 mg mL^−1^) in DMSO-d_6_ and transferred into an NMR tube. ^1^H-NMR spectra were recorded in solvent suppression mode at 298 K using a Bruker AVIII 400 MHz BBO NMR spectrometer (Bruker Corporation, Coventry, UK). The ^1^H-NMR spectra acquired were phased, baseline-corrected, and integrated using Bruker Topspin 4.2.0 software. The monomer conversion (MC) was calculated using the relative area of the doublet of doublets (*dd*) of the Z-β-protons of NIPAm (δ ~ 5.55), MBA (δ ~ 5.66 ppm), NAPMAm (δ ~ 5.36 ppm), and NHMAm (δ ~ 5.64 ppm) against the single peak of the aryl protons (δ = 6.87 ppm) of the IS at times 0 and 24 h, as described in Equation (1). Total MC was obtained by the sum of each individual monomer concentration multiplied by its molar fraction:

MC = 100 − (100 × A_t = 24h_)⁄A_t = 0 h_, (1)

where MC is the monomer conversion (molar%), and *A_t_*_= 24 h_ and *A_t_*_= 0 h_ are the relative areas of the corresponding peaks against the TMB after 24 h and 0 h of polymerisation, respectively.

#### 2.3.2. Size Measurements via DLS

The hydrodynamic diameter (*D_h_*) of the microgels was measured by DLS using the Zetasizer Nano ZS fitted with a 4 mW He-Ne 633 nm laser module and operated with the software ZS Xplorer (version 1.5.0.163) (Malvern Instruments Ltd., Malvern, UK). All samples were analysed as dispersions in deionised water (0.5 mg mL^−1^) after 24 h of swelling and filtration through a 1.0 µm PES filter (Fisher Scientific, Loughborough, UK). Analyses were performed using a disposable cuvette (Fisher Scientific, Leicestershire, UK, catalogue no. 15520814), at 25 °C, with a detection angle of 173° (back-scattering). Material method was set to polymer latex, and data were obtained by cumulative analyses of the correlation function using the Stokes–Einstein equation. The data are presented as mean values of *D_h_* based on number distribution calculated over three measurements.

#### 2.3.3. VPTT Experiments

The VPTT values of the microgels were obtained using a Cary 100 UV–vis spectrophotometer equipped with an internal temperature controller (Agilent Technologies, Cheshire, UK). All samples were analysed as stable colloidal solutions in deionised water (1.0 mg mL^−1^) after 24 h of swelling and filtration through a 1.0 µm PES filter. The measurements were performed using high-precision Suprasil quartz cuvettes (optical path = 10 mm) (Hellma, Southend-on-Sea, UK) at the fixed excitation wavelength (*λ_ex_*) of 500 nm. A temperature scanning from 20 °C to 60 °C at a rate of 0.5 °C min^−1^ was performed to assess the absorbance shift along with the temperature increase. Absorbance readings were recorded every minute for each temperature point, and absorbance data for each sample were blank-corrected and converted in transmittance (%) using Equation (2):

T = 10^2−A^, (2)

where *T* is the transmittance (%) and *A* is the absorbance (%). The data were fitted to the Boltzmann model using the OriginPro 9.0 software (OriginLab Corporation, Northampton, MA, USA). The VPTT was defined as the single-point temperature at which the transmittance dropped to 50%.

To assess the thermoresponsive behaviour of microgels by DLS, *D_h_* of each sample was measured (triplicate, backscatter angle mode) every 2 °C from temperatures ranging from 20 to 60 °C, allowing 10 min equilibration time at every temperature change.

## 3. Results and Discussion

**Figure 1:**
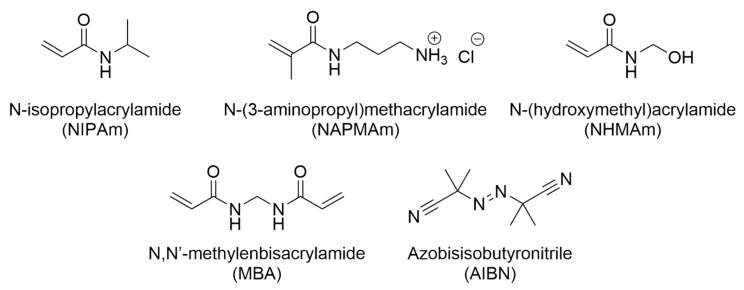
Chemical structure of the backbone monomer (NIPAm), functional co-monomers (NAPMAm and NHMAm), crosslinker (MBA), and initiator (AIBN) used to synthesise the microgels.

A library of NIPAm-based microgels, covalently crosslinked with MBA, was prepared by free radical polymerisation in water (Figure 1). NIPAm was chosen as the backbone monomer as it forms one of the most widely studied thermoresponsive polymeric networks, in addition to having VPTT values that are physiologically relevant [46]. Previous work showed that NIPAm-based microgels crosslinked with MBA resulted in particles that could be identified by a core–shell structure [47,48], while a lower crosslinking content yielded more swellable, and potentially larger, particles [49]. In this work, the crosslinking content in the formulations was varied between 2 and 10 molar% to allow the study of its role in influencing particle size and its relationship to the other experimental variables, e.g., total monomer concentration and presence of functional co-monomers.

A key advantage of free radical polymerisation is the ability to easily tailor the microgels’ composition by changing their formulation, without variations in the synthetic methodology. In this work, functional co-monomers NHMAm and NAPMAm (Figure 1) were added to the formulations to introduce primary alcohols groups, providing hydrogen bonding, and primary amine groups, that under physiological conditions would result in positive charges, respectively. Additionally, both functional co-monomers share a structural similarity to NIPAm, with the polymerizable acrylamide-based unit, and this is expected to limit any significant differences in the reaction rates during the polymerisation.

Previous studies provided evidence that the choice of the initiator can impact the final composition of the microgels [7]. In this work, AIBN was chosen as the initiator for the polymerisation, as it does not introduce additional charges to the microgel [7], limiting the variability when functional monomers are added to the formulations. It was previously reported that the polymerisation of NIPAm-based microgels with AIBN requires temperatures above 60 °C to achieve suitable yields [48]. Given that NIPAm is thermoresponsive and has limited solubility in aqueous solutions at 60 °C, the synthesis required the presence of CTAB as surfactant to maintain the colloidal stability of the growing polymeric chains [50].

### 3.1. Effect of Total Monomer Concentration (C_m_) on Particle Size

Given our drive towards the development of sustainable synthetic methodologies [30], the first step focused on the synthesis in water of NIPAm-based microgels with MBA content at 2, 5, and 10 molar%, to provide reference data for the other formulations (Table S1). When comparing previously reported data on the synthesis in dimethylsulfoxide, the microgels prepared in water were obtained with higher MC values (>99 molar%) and higher chemical yields (>81 wt%) [28]. The difference between MC values and yields can be attributed to the loss of short polymer chains during the purification step via dialysis. The combination of these two variables ensured a high consistency between the feeding formulation and the final chemical composition, a key priority given the random nature of radical polymerisation, that does not allow to determine the exact structure of the polymers. The *D_h_* and polydispersity indexes (PDIs) of the isolated microgels were measured in aqueous dispersions at 0.5 mg mL^−1^, using DLS in back-scattering mode. The data show that the change in crosslinker content had limited effect on size (measured by number), ranging from 108 nm for 2 molar% MBA to 134 nm for 10 molar% MBA (Figure S2). The data are supported by good-quality correlation functions (Figure S2 inserts) and low PDI values, with these two parameters used throughout this work to evaluate data on the particles’ sizes. TEM images were not acquired, as drying of the samples on a grid would be required, resulting in much lower sizes, as extensively discussed in the work of Khutoryaskiy et al. [51]. Given the low dispersity of the polymers, the conversion of the intensity-weighted distribution into the number-weighted distribution was deemed suitable, as this is reported to produce values much closer to the ones identified via TEM.

**Figure 2:**
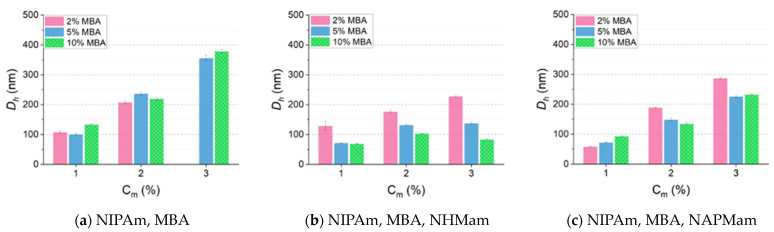
Hydrodynamic sizes measured by DLS (data by number; 0.5 mg mL^−1^ in water) for microgels prepared with different crosslinker content (MBA, 2% to 10%) and at varying C_m_: (**a**) neutral microgels (MG-X_x_), (**b**) microgels containing hydrogen-bonding groups (MG-X_x_O_5_), and (**c**) microgels containing amine-charged groups (MG-X_x_N_5_). Data for MG-X_2_ at C_m_ 3 wt% not reported as polymer did not form a monodisperse stable colloidal solution.

The impact of changing the C_m_ to tailor *D_h_* was initially evaluated for neutral microgels prepared in water with NIPAm and varying crosslinking (MBA) degrees (2, 5, and 10 molar%). The results shown in Figure 2a (correlograms in Figure S3) provide evidence of the key role played by C_m_ in determining the *D_h_* for all formulations. The *D_h_* of microgels prepared with 2 molar% MBA and C_m_ equal to 1 or 2 wt% was 108 and 208 nm, respectively, resulting in an increase of 1.9-fold in *D_h_*. A similar trend was observed for microgels prepared with 5 and 10 molar% of MBA, where the *D_h_* of microgels increased by 3.5- and 2.8-fold, respectively. With the formulation prepared with 2 molar% MBA at C_m_ 3 wt%, the *D_h_* could not be obtained reliably, as the formulation was not monodispersed nor colloidally stable. These results are consistent with literature data [52] and can be explained by the more frequent collisions, resulting from higher C_m_, during the polymerisation.

Interestingly, when considering the impact on *D_h_* as a result of varying the crosslinker content in the formulations, the data suggest that any variation is minimal when compared to the effect resulting from changing the C_m_. Small variations were observed, for example, between 2 and 5 molar% MBA at C_m_ 2 wt%, going from 208 nm to 237 nm; however, these were not considered to be sufficiently significant when the standard deviation was taken into account. In the next step, we evaluated the effect on the size of microgels of introducing specific functional groups.

### 3.2. The Role of Hydrogen Bonding in Controlling Particle Size

Hydrogen bonds are important intermolecular forces for all biological systems [53]; however, the addition of hydrogen-bonding groups has the potential to greatly interfere in the outcome of polymerisation in water due to the interactions with the solvent. To evaluate the impact of adding functional groups capable of hydrogen bonding to the microgels’ properties, the monomer NHMAm, containing primary alcohol groups, was included in the formulation. To minimise changes in the reaction kinetics and highlight the sole effect of the hydrogen-bonding groups on the free radical polymerisation, NHMAm was added at a concentration of 5 molar%.

All microgels prepared with the addition of NHMAm showed quantitative MC (>99 molar%) and yields above 82 wt% (Table S2), displaying good consistency between the feeding solution and the final chemical composition. DLS was used to obtain *D_h_* (correlograms in Figure S4, data on size by intensity and PDI in Table S2). The addition of NHMAm to the heterogeneous polymerisation of NIPAm-based microgels resulted overall in the formation of smaller particles, when compared to the non-functionalised microgels (Figure 2b), with changes in C_m_ and MBA content appearing to have a significant impact, in stark contrast to what was observed for the unfunctionalised polymers. When compared to non-functionalised microgels, the variation in C_m_ resulted in a less significant change in *D_h_*, with the polymer with 2 molar% MBA displaying only a 1.8-fold increase when going from C_m_ 1 wt% (129 nm) to C_m_ 3 wt% (228 nm). On the other hand, the flexibility of the matrix appeared to be a more important factor influencing the *D_h_*. For instance, for MG-X_x_O_5_ prepared with C_m_ 3 wt%, the increase in crosslinker content from 2 to 10 molar% resulted in a significant drop in *D_h_* from 228 nm to 84 nm. Overall, the results provide evidence that the addition of hydrogen-bonding groups allows a precise control of *D_h_* by changing C_m_ and crosslinker content, being useful tools to control particle size.

### 3.3. The Role of Charge in Controlling Particle Size

To further investigate the role of functional groups on particle size, NAPMAm was added to NIPAm-based microgels to introduce positive charges to the polymeric structure at physiological conditions. Being the strongest noncovalent interaction [54], electrostatic interactions are widely explored for biomedical applications [55,56,57,58], as the presence of charge can target specific tissues or organs. NAPMAm has a pKa ~ 9.0, and it is fully ionised in the reaction mixture, being a suitable model to investigate the role of electrostatic interactions on *D_h_*. Again, the functional co-monomer was added at a concentration of 5 molar% to avoid a dramatic change in polymerisation kinetics.

All microgels prepared with the addition of NAPMAm showed quantitative MC (>99 molar%) and yields above 72 wt% (Table S2), showing consistency between the feeding solution and the final chemical composition. The impact of increasing C_m_ from 1 to 3 wt% was more similar to what was observed with neutral microgels (size by number in Figure 2c, correlograms in Figure S5, and size by intensity and PDI in Table S2), with the more flexible polymers, containing 2 molar% MBA, showing a 4.9-fold increase (59 nm to 287 nm), although even at 10 molar% MBA the change in *D_h_* was very significant (2.5-fold increase, from 94 nm to 233 nm). These data suggest that the presence of the positively charged groups influence a lot more the effect that alterations in the formulation can have on *D_h_*, with important implications for applications. When quantifying the impact of changing the crosslinker content at the different C_m_, the results do not provide such a clear trend: at C_m_ 1 wt% the monomers are quite diluted, and an increase in crosslinker content from 2 to 10 molar% MBA lead to larger particles (*D_h_* of 59 nm and 94 nm, respectively). However, when the C_m_ was doubled to 2 wt%, the increased rigidity of the matrix results in a significant drop in particle size, going from 189 nm to 135 nm. A possible explanation for this difference relates to the fact that, as the crosslinker content and C_m_ increases, the particles are likely to form a more dense core, with a smaller hydration shell [59]. The data suggest that the C_m_ used for the polymerisation need to be carefully chosen, as the effect of crosslinker content on *D_h_* can fluctuate considerably.

The systematic synthesis of a library of NIPAm-based microgels allowed to evaluate the role of the crosslinking content, the C_m_, and the presence of functional groups on the *D_h_*. The C_m_ had an impact on the *D_h_* of neutral, hydrogen-bonding, and cationic microgels, although more significant on neutral non-functionalised microgels (Figure 2a). The crosslinking degree played an important role in influencing *D_h_*, especially for microgels containing hydrogen-bonding monomers, where a clear trend can be observed between MBA content and *D_h_* for all formulations prepared with C_m_ above 2 wt% (Figure 2b). The magnitude of the effect of functional groups on the *D_h_* was better observed for microgels prepared with 10 molar% of MBA and C_m_ 3%: neutral, hydrogen-bonding, and cationic microgels measured 379 nm, 84 nm, and 233 nm, respectively. The data overall demonstrate that functional monomers with their side groups can be powerful tools to tailor microgel size when prepared via heterogeneous polymerisation in water.

### 3.4. Impact of Crosslinking Degree and Functional Groups on the Thermoresponsive Properties of NIPAm-Based Microgels

The control of the thermoresponsive properties of NIPAm-based microgels is essential for multiple applications, so the effect of the crosslinking content and functional co-monomers on the thermoresponsive behaviour of NIPAm-based microgels was investigated via studies to determine the VPTT [60]. As it was previously shown that VPTT has a linear relation with size, microgels with similar sizes were selected for this study to ensure that the analysis was focused on only one variable (Table 1).

**Figure 3:**
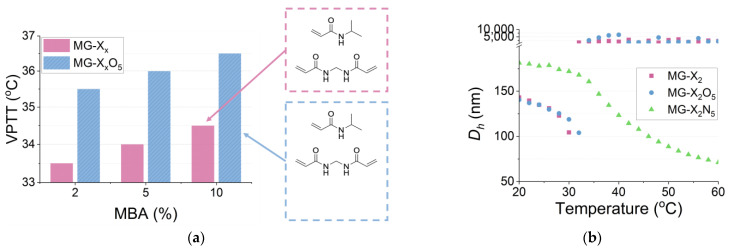
Volume phase transition temperature (VPTT) data obtained for the selected microgels by (**a**) UV–vis spectrophotometry (1.0 mg mL^−1^ in deionised water, temperature range of 20–50 °C, heating rate of 0.5 °C min^−1^) and (**b**) dynamic light scattering (0.5 mg mL^−1^ in deionised water, back-scattering, temperature range of 20–60 °C, heating rate of 0.2 °C min^−1^). *D_h_* = hydrodynamic size.

The thermoresponsive properties of microgels, dispersed in deionised water at 1 mg mL^−1^, were evaluated using UV–vis spectrophotometry. The data highlight the impact of the different formulations on VPTT values (Figure 3a). The crosslinking degree played a crucial role in the VPTT for neutral and hydrogen-bonding microgels, where the increase in rigidity resulted in higher VPTT values (Figure S6). Adding hydrogen-bonding groups led to an increase of 2 °C to the VPTT of microgels, resulting in a range (35.5–36.5 °C) that is more relevant, for example, to biomedical applications. The increase in hydrophilicity resulting from the addition of NHMAm restrains the desolvation of water molecules, hindering the structural change involved in the thermoresponsive property of NIPAm [61]. The thermoresponsive behaviour was also evaluated using DLS in the temperature range of 20–60 °C, and the data (Figure 3b) show aggregation for both MG-X_x_ and MG-X_x_O_5_ at temperatures above 32 and 34 °C, respectively.

Cationic microgels (MG-X_x_N_5_) showed thermostability up to 60 °C, and no VPTT could be determined by UV–vis spectrophotometry in the temperature range evaluated. However, the thermoresponsive effect was confirmed by DLS, where it was possible to observe a decrease of 28% in *D_h_* when increasing the temperature from 30 to 40 °C (Figure 3b). This behaviour is consistent with the results from the ζ-potential (Table 1), that confirmed the presence of positive charges on the surface that are likely to stabilise the particles via charge repulsion.

The data on the thermoresponsive properties of the microgels provide evidence of the important role that hydrogen-bonding groups and positive charges have in the temperature-driven conformational changes of NIPAm-based microgels. Therefore, while being valuable tools to tailor *D_h_*, the addition of functional co-monomers must consider the effect on the thermoresponsive properties of the microgels, as those have a great influence on their application.

## 4. Conclusions

Controlling the size of microgels is important for multiple applications, as it is closely linked to key physico-chemical properties. Functional groups bearing hydrogen-bonding capabilities or positive charges have been extensively studied, in particular for drug-uploading application. However, little is known about the effect of adding such functional monomers to the formulation and how it will impact the *D_h_* of microgels, when prepared by free radical polymerisation in water. We investigated the effect of C_m_, crosslinking degree, and functional groups on the size and thermoresponsive properties of microgels synthesised in water. Synthesis in water resulted in monodisperse microgels and allowed the control of *D_h_* by changing C_m_ and MBA content. Across all of the formulations, the C_m_ played a significant role in controlling *D_h_*, and the increase in C_m_ from 1 to 3 wt% resulted in an increase in size up to 4.9-fold (MG-X_2_N_5_). The crosslinking content allowed the fine-tuning of microgel size, especially for microgels with hydrogen-bonding groups (MG-X_x_O_5_), where the increase in matrix rigidity resulted in a decrease in *D_h_*. The influence of functional co-monomers was more evident at higher C_m_, where the absolute difference in *D_h_* varied greatly between formulations. As expected, introducing hydrogen-bonding monomers increased the VPTT by 2 °C, when compared to the neutral non-functionalised microgels. The addition of positive charges generated particles that showed a decrease in size upon heating but did not aggregate at temperatures up to 60 °C. In conclusion, the microgel formulation must be carefully tailored to the desired application, as to ensure adequate *D_h_* and thermoresponsive properties. In this work, we broadened the knowledge of the effect of synthetic conditions and formulation on the *D_h_* and thermoresponsive properties. However, as *D_h_* is a crucial property for the application of nanomaterials, synthetic conditions must be optimised during the development phase of any industrial application.

## Figures and Tables

**Table 1:** Formulation of microgels, with data on size and ζ-potential determined by dynamic light scattering (DLS), and volume phase transition temperature (VPTT) determined by UV–vis spectrophotometry.

Code	NIPAm (molar%)	NAPMAm (molar%)	NHMAm (molar%)	MBA (molar%)	Size by Intensity (nm)	Size by Number (nm)	PDI	ζ-Potential (mV)	VPTT (°C)
MG-X_2_	98	0	0	2	168	108	0.165	-	33.5
MG-X_5_	95	0	0	5	147	114	0.036	-	34.0
MG-X_10_	90	0	0	10	162	134	0.020	-	34.5
MG-X_2_O_5_	93	0	5	2	197	149	0.067	-	35.5
MG-X_5_O_5_	90	0	5	5	193	132	0.026	-	36.0
MG-X_10_O_5_	85	0	5	10	141	104	0.019	-	36.5
MG-X_2_N_5_	93	5	0	2	246	189	0.143	+3.9	-
MG-X_5_N_5_	90	5	0	5	194	149	0.101	+5.0	-
MG-X_10_N_5_	85	5	0	10	185	135	0.096	+7.0	-

## Data Availability

The dataset is available on request from the authors.

## Supplementary Materials

The following supporting information can be downloaded at https://www.mdpi.com/article/10.3390/polym16243532/s1: Table S1. Library of microgels prepared in water with different monomer concentrations (C_m_) and crosslinking degrees, showing their composition, chemical yields, monomer conversion (MC) values, and size measurements. MC values were obtained by ^1^H-NMR, and size measurements (with corresponding standard deviations) were obtained by dynamic light scattering (DLS) in back-scattering mode (*n* = 3). PDI = polydispersity index; Table S2. Library of microgels prepared in this study, showing their composition, chemical yields, monomer conversion (MC) values, size measurements, and volume phase transition temperature (VPTT) data. All microgels were prepared with 1.7 molar% AIBN (initiator) and 0.6 mg mL^−1^ of CTAB (surfactant). MC values were obtained by ^1^H-NMR, and size measurements (with corresponding standard deviations) were obtained by dynamic light scattering (DLS) in back-scattering mode (*n* = 3). PDI = polydispersity index; Figure S1: Example of ^1^H-NMR spectra of a NIPAm-MBA feeding solution before (t = 0 h) and after (t = 24 h) the free radical polymerisation. The chemical structures and the tracked protons are highlighted in corresponding colours; Figure S2. Hydrodynamic size (*D_h_*) distributions by intensity, number, and volume of microgels by dynamic light scattering (DLS). Inserts show the corresponding correlograms. Microgel dispersions in deionised water were analysed in triplicate at 0.5 mg mL^−1^ in back-scattering mode. (a) MG-X_2_, (b) MG-X_5_, and (c) MG-X_10_. PDI values were 0.165, 0.029, and 0.020, respectively; Figure S3. Correlograms of neutral microgels (MG-X_x_) obtained by dynamic light scattering (DLS). Microgel dispersions in deionised water were analysed in triplicate at 0.5 mg mL^−1^ in back-scattering mode; Figure S4. Correlograms of microgels containing hydrogen-bonding groups (MG-X_x_O_5_) obtained by dynamic light scattering (DLS). Microgel dispersions in deionised water were analysed in triplicate at 0.5 mg mL^−1^ in back-scattering mode; Figure S5. Correlograms of amine-charged microgels (MG-X_x_N_5_) obtained by dynamic light scattering (DLS). Microgel dispersions in deionised water were analysed in triplicate at 0.5 mg mL^−1^ in back-scattering mode; Figure S6. Volume phase transition temperature (VPTT) data obtained by UV–vis spectrophotometry of microgels (a) MG-X_x_ and (b) MG-X_x_O_5_. Measurements were performed with microgels’ dispersions in deionised water at 1.0 mg mL^−1^, temperature range of 20–50 °C, and heating rate of 0.5 °C min^−1^.
