# Supplementary material for: NIPAm Microgels Synthesised in Water: Tailored Control of Particles’ Size and Thermoresponsive Properties
Source: Polymers (Basel). 2024 Dec 18;16(24):3532. doi: 10.3390/polym16243532 (PMC11679721; doi:10.3390/polym16243532)
Supplement: Supplementary file 1 [file polymers-16-03532-s001.zip › polymers-3357235-supplementary.pdf]

Electronic Supplementary material

NIPAm microgels synthesised in water: tailored control of particles' size and thermoresponsive properties

Gabriela Rath,[a] Davide Mazzali,[a] Ali Zarbakhsh,[a] and Marina Resmini *[a]

[a.] *School of Physical & Chemical Sciences, Queen Mary University of London, Joseph Priestley Building, Mile End Road, London E1 4NS, UK.*
** E-mail: m.resmini@qmul.ac.uk*

Supporting tables

Table S1. Library of microgels prepared in water with different monomer concentrations (C_m) and crosslinking degrees, showing their composition, chemical yields, monomer conversion (MC) values, and size measurements. MC values were obtained by ^{1}H NMR, and size measurements (with corresponding standard deviations) were obtained by dynamic light scattering (DLS) in back-scattering mode (*n*=3). PDI = polydispersity index.

Code	C_m (wt%)	T (ºC)	NIPAm (molar%)	MBA (molar%)	AIBN (molar%)	CTAB (mg mL^{-1})	Yield (wt%)	MC (molar%)		Size by intensity (nm)	Size by number (nm)	PDI
								NIPAm	MBA			
**MG-X$_2$C$_1$**	1	70	98	2	1.7	0.6	87.8	>99	>99	168±2 (100%)	108±3 (100%)	0.165
**MG-X$_2$C$_2$**	2	70	98	2	1.7	0.6	83.0	>99	>99	249±4 (100%)	208±5 (100%)	0.072
**MG-X$_2$C$_3$**	3	70	98	2	1.7	0.6	87.7	>99	>99	N/A*	N/A*	too high*
**MG-X$_5$C$_1$**	1	70	95	5	1.7	0.6	89.6	>99	>99	131±3 (100%)	101±3 (100%)	0.029
**MG-X$_5$C$_2$**	2	70	95	5	1.7	0.6	94.5	>99	>99	267±6 (100%)	237±6 (100%)	0.020
**MG-X$_5$C$_3$**	3	70	95	5	1.7	0.6	82.1	>99	>99	400±9 (100%)	356±9 (100%)	0.014
**MG-X$_{10}$C$_1$**	1	70	90	10	1.7	0.6	84.6	>99	>99	162±2 (100%)	134±2 (100%)	0.020
**MG-X$_{10}$C$_2$**	2	70	90	10	1.7	0.6	81.7	>99	>99	250±4 (100%)	220±5 (100%)	0.012
**MG-X$_{10}$C$_3$**	3	70	90	10	1.7	0.6	75.4	>99	>99	419±9 (100%)	379±6 (100%)	0.034

* For C_m 3 wt% the formulation did not provide a stable monodisperse colloidal solution, with PDI>0.4.

Table S2. Library of microgels prepared in this study, showing their composition, chemical yields, monomer conversion (MC) values, size measurements, and volume phase transition temperature (VPTT) data. All microgels were prepared with 1.7 molar% AIBN (initiator) and 0.6 mg mL^{-1} of CTAB (surfactant). MC values were obtained by ^{1}H NMR, and size measurements (with corresponding standard deviations) were obtained by dynamic light scattering (DLS) in back-scattering mode (*n*=3). PDI = polydispersity index.

Code	NIPAm (molar%)	NAPMAm (molar%)	NHMAm (molar%)	MBA (molar%)	C_m (wt%)	T (ºC)	Yield (wt%)	MC (molar%)	Size by intensity (nm)	Size by number (nm)	PDI	VPTT (ºC)	ζ-potential (mV)
MG-X_2	98	0	0	2	1	70	88	>99	168±3	108±3	0.165	33.5	0
MG-X_5	95	0	0	5	1	70	90	>99	131±3	101±3	0.029	34.0	0
MG-X_{10}	90	0	0	10	1	70	82	>99	162±2	134±2	0.020	34.5	0
MG-X_2O_5	93	0	5	2	2	70	82	>99	237±1	177±4	0.192	35.5	0
MG-X_5O_5	90	0	5	5	2	70	90	>99	193±1	132±3	0.280	36.0	0
MG-$X_{10}O_5$	85	0	5	10	2	70	89	>99	168±2	104±1	0.019	36.5	0
MG-X_2N_5	93	5	0	2	2	70	73	>99	246±2	189±1	0.143	-	+3.9
MG-X_5N_5	90	5	0	5	2	70	74	>99	194±3	149±5	0.101	-	+5.0
MG-$X_{10}N_5$	85	5	0	10	2	70	72	>99	185±2	135±3	0.096	-	+7.0

Supporting figures

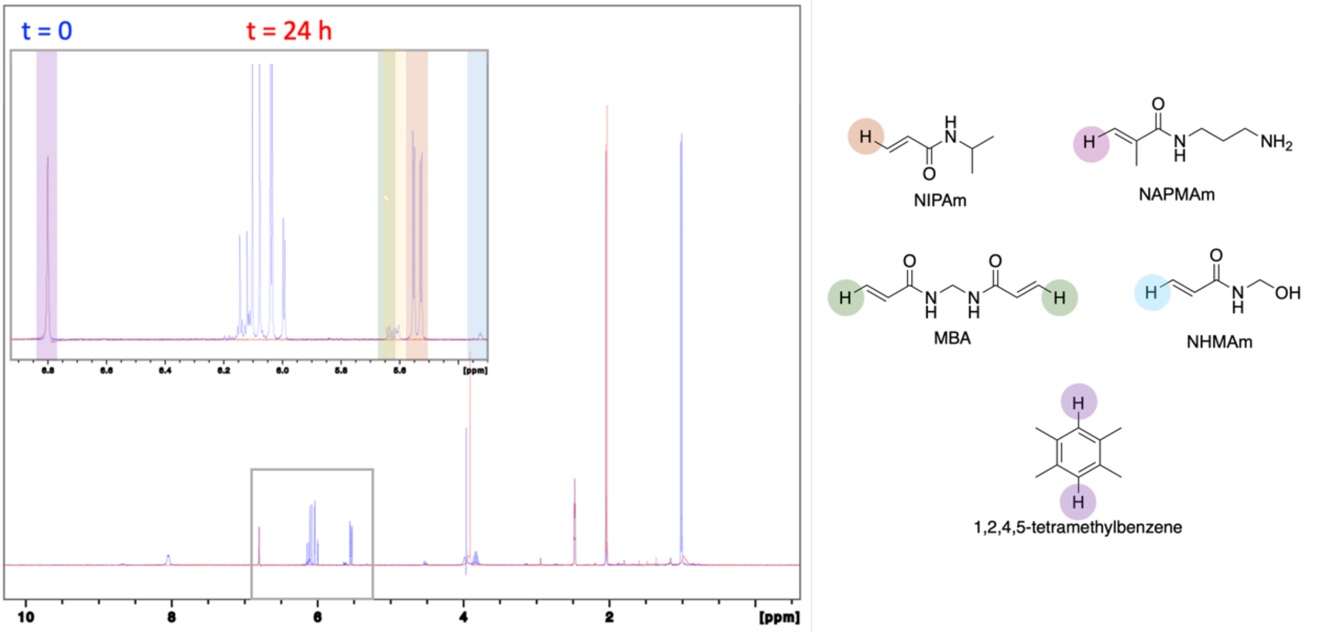

Figure S1. Example of a ^{1}H-NMR spectra of a NIPAm-MBA feeding solution before (t=0h) and after (t=24h) the free radical polymerization. The chemical structures and the tracked protons are highlighted in corresponding colours.

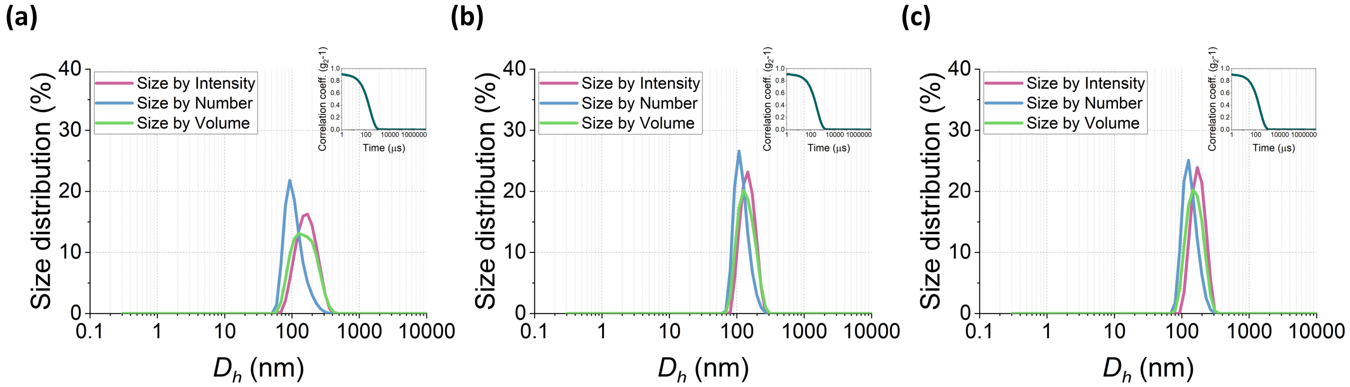

Figure S2. Hydrodynamic size (D_h) distributions by intensity, number, and volume of microgels by dynamic light scattering (DLS). Inserts show the corresponding correlograms. Microgel dispersions in deionised water were analysed in triplicate at 0.5 mg mL^{-1} in back-scattering mode. **(a)** MG-X_2, **(b)** MG-X_5 and **(c)** MG-X_{10}. PDI values were 0.165, 0.029 and 0.020 respectively.

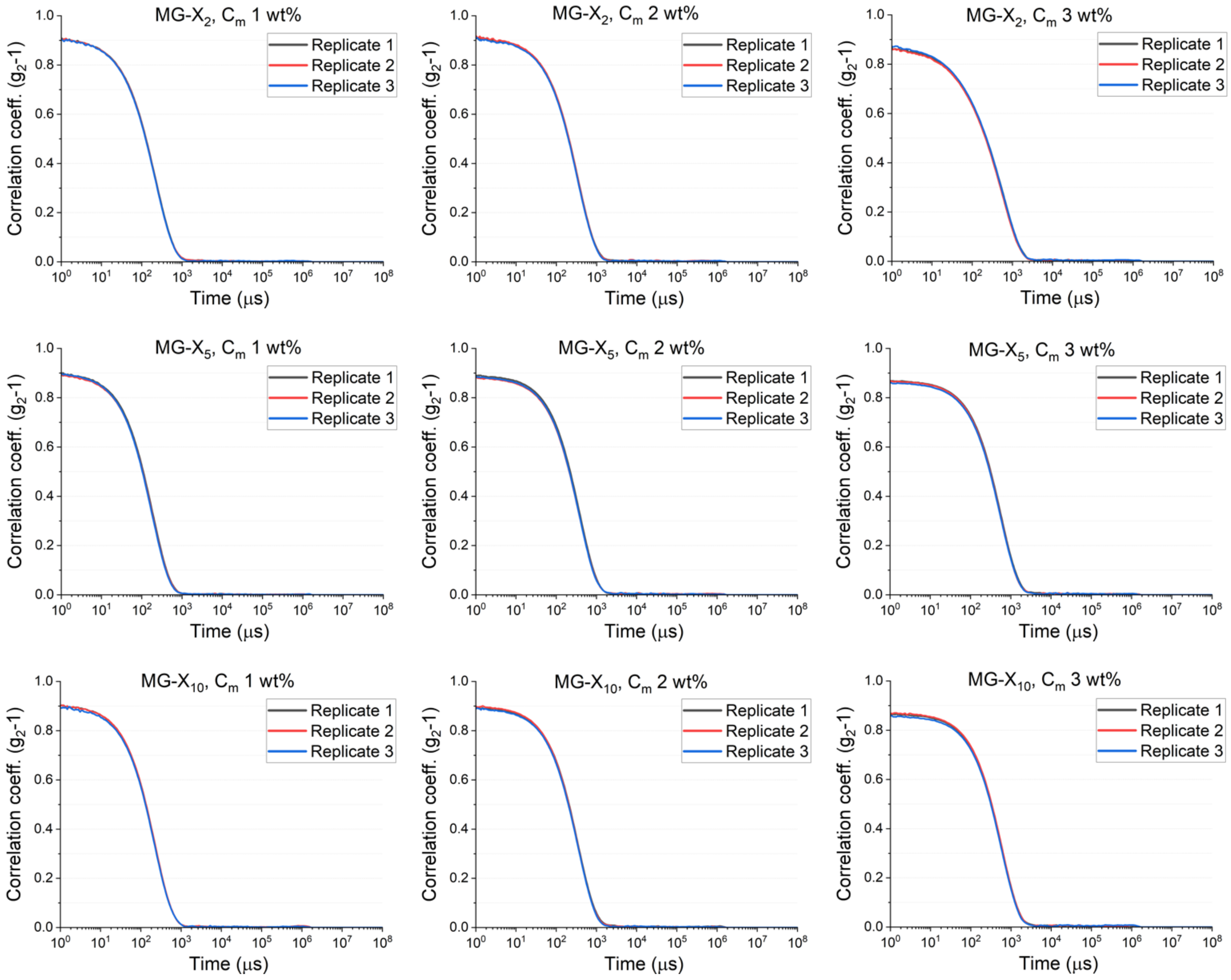

Figure S3. Correlograms of neutral microgels (MG-X_x) obtained by dynamic light scattering (DLS). Microgel dispersions in deionised water were analysed in triplicate at 0.5 mg mL^{-1} in back-scattering mode.

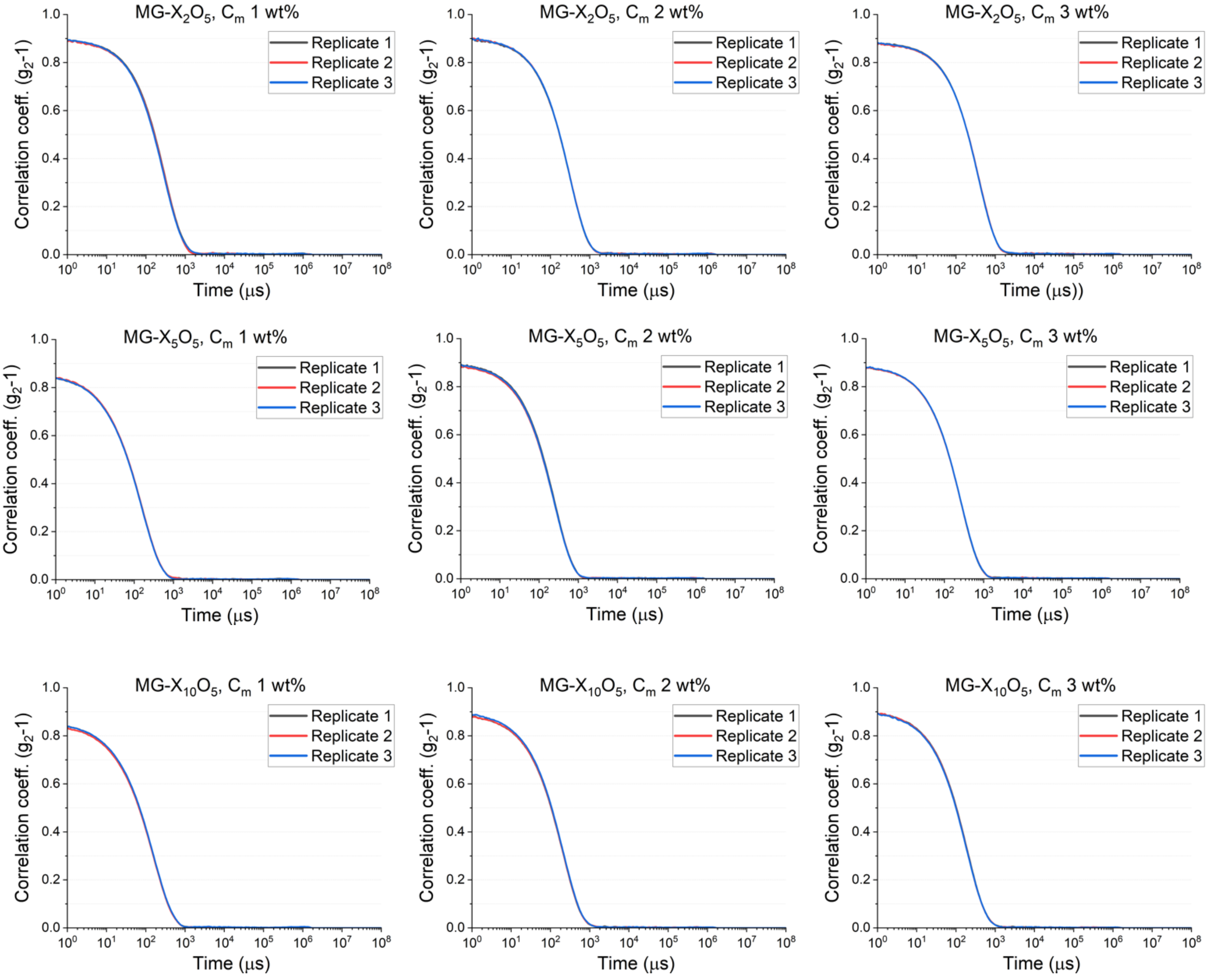

Figure S4. Correlograms of microgels containing hydrogen bonding groups (MG-X_xO_5) obtained by dynamic light scattering (DLS). Microgel dispersions in deionised water were analysed in triplicate at 0.5 mg mL^{-1} in back-scattering mode.

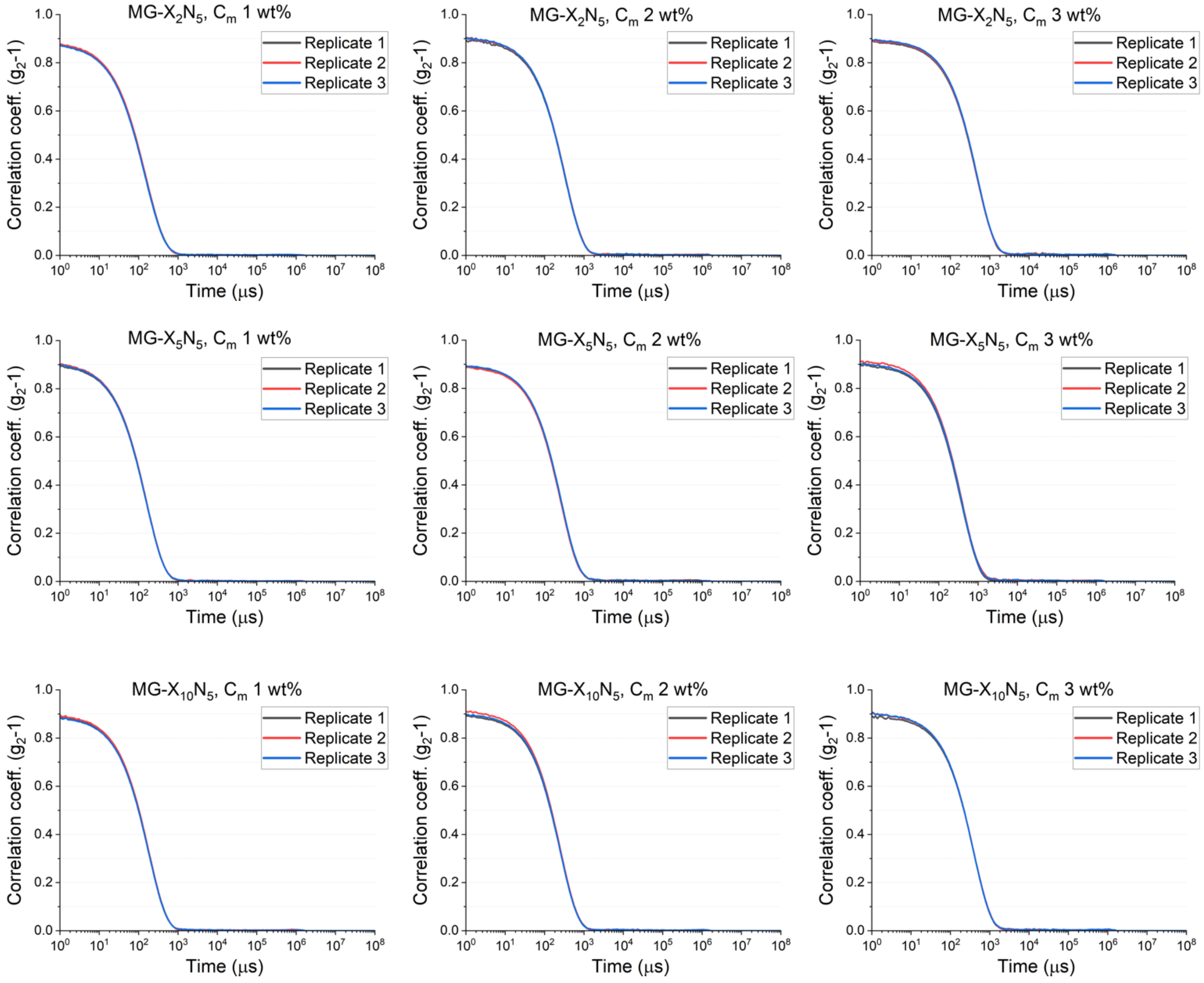

Figure S5. Correlograms of amine-charged microgels (MG-X$_x$N$_5$) obtained by dynamic light scattering (DLS). Microgel dispersions in deionised water were analysed in triplicate at 0.5 mg mL^{-1} in back-scattering mode.

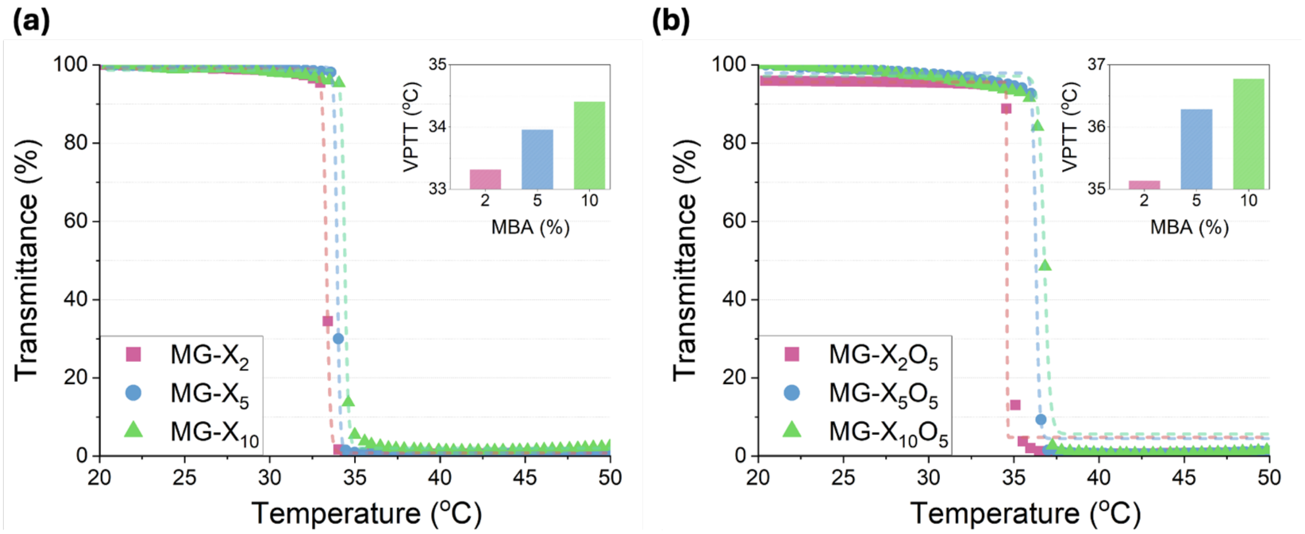

Figure S6. Volume Phase Transition Temperature (VPTT) data obtained by UV-*vis* spectrophotometry of microgels **(a)** MG-X$_x$ and **(b)** MG-X$_x$O$_5$. Measurements were performed with microgels dispersions in deionised water at 1.0 mg mL^{-1}, temperature range of 20-50 ºC, and heating rate of 0.5 ºC min^{-1}.
